# Down-regulation of microRNA-155 promotes selenium deficiency-induced apoptosis by tumor necrosis factor receptor superfamily member 1B in the broiler spleen

**DOI:** 10.18632/oncotarget.17222

**Published:** 2017-04-19

**Authors:** Ci Liu, Zhepeng Sun, Zhe Xu, Tianqi Liu, Tingru Pan, Shu Li

**Affiliations:** ^1^ College of Veterinary Medicine, Northeast Agricultural University, Harbin 150030, P. R. China

**Keywords:** broiler, spleen, lymphocyte apoptosis, microRNA-155, tumor necrosis factor receptor superfamily member 1B

## Abstract

The aim of this work was to explore the microRNA profile and the effect of microRNA-155 on apoptosis in the spleen of selenium-deficient broilers. We replicated the splenic-apoptotic model in selenium-deficient broilers. *In vitro*, microRNA-155 oligonucleotides were transfected into lymphocytes and subsequently treated with H_2_O_2_. We observed that selenium deficiency altered the microRNA profile and decreased the expression of microRNA-155 in the broiler spleens. Tumor necrosis factor receptor superfamily member 1B was verified as a target of microRNA-155 in the splenocytes. Morphological changes, increased levels of tumor necrosis factor receptor superfamily member 1B, c-Jun N-terminal kinase, Bak, Bax, Cyt-c, caspase9 and caspase3 and decreased levels of Bcl-2 demonstrated that selenium deficiency induced apoptosis in the spleen tissues. *In vitro*, microRNA-155 m inhibited the levels of ROS and reduced apoptosis compared with microRNA-155i in the lymphocytes. These results suggested that the reduced levels of microRNA-155 due to selenium deficiency could promote oxidative stress-induced apoptosis by increased tumor necrosis factor receptor superfamily member 1B in splenic cells.

## INTRODUCTION

The essential trace mineral selenium is of fundamental importance in animal health. Selenium plays a crucial role in immunomodulation [1]. Selenium supplementation can improve the immune function of animals [2]. Selenium modulates the immune response by protecting lymphocytes from the effects of inhibitory products and maintaining the integrity of cellular membranes [3]. However, selenium deficiency or low-selenium status may negatively affect immune cells, such as reducing the maturation of specific subpopulations and proliferative capabilities of lymphocytes; blocking biosynthesis in macrophages and decreasing the anti-oxidation and bactericidal ability of neutrophils [4–6]. Selenium deficiency also impaired the host innate immune response by inducing changes in the levels of cytokine expression in the spleens of mice [7]. Furthermore, selenium deficiency aggravates the caspase3-dependent apoptosis induced by H_2_O_2_ in primary cultures of pig thyroid cells [8]. The caspase-dependent apoptotic pathway was up-regulated by selenium deficiency in myocardial of rats [9]. Selenium deficiency induced apoptosis and linked the oxidative and endoplasmic reticulum stress pathways in the skeletal muscles (wing, pectoral, and thigh) and livers of laying hens [10, 11]. Apoptosis linked to nitric oxide was induced by selenium deficiency in the immune organs of laying hens [12].

MicroRNAs are a class of short (21–25 nt), non-coding RNAs that are evolutionarily conserved from plants to mammals and they control the expression of their target genes at the post-transcriptional level [13]. Apoptosis is an intrinsic cellular mechanism and can be stimulated by various microRNAs. miRs function as either pro- or anti-apoptotic factors mainly by binding to the 3'-untranslated region (3'UTR) of their target mRNA resulting in mRNA cleavage and/or translational inhibition to prevent the expression of the corresponding protein [14]. miR-155 represents a typical multifunctional microRNA in the immune system that affects cell activation, proliferation and differentiation [15–18]. Available experimental evidence demonstrates that microRNA-155 can mediated inhibition of apoptosis by interacting with its downstream targets in various cell lines [19, 20]. Furthermore, the selenium supply can alter the microRNA expression profile. Microarray analysis (737 microRNAs in total) of the microRNA profile of Caco-2 cells growing in selenium-deficient or selenium-supplemented medium revealed that the expression of 12 microRNAs were affected by the selenium supply [21]. Among the 621 microRNAs detected by GeneChip, microRNA222, microRNA2393, and microRNA2300b were significantly affected by selenium supplementation in the liver tissues of maturing beef [22].

In summary, selenium deficiency could cause immune dysfunction and damages to the immune organs in chickens. In this study, our aim was to investigate the effects of selenium deficiency on the microRNA profile and the mechanism of microRNAs in apoptosis of splenic cells. Therefore, we successfully reproduced the model of selenium-deficient broilers. We found that selenium deficiency altered the microRNA profile and reduced the expression of microRNA-155 in the tissues of the spleen. In addition, tumor necrosis factor receptor superfamily member 1B (TNFRSF1B) was predicted as one of the microRNA-155 targets in broilers. Based on the pivotal role of TNFRSF1B in the apoptotic pathway, we performed experiments both *in vivo* and *in vitro*. The results demonstrated that TNFRSF1B was a target of microRNA-155 in the splenic cells. Reduced levels of microRNA-155 promoted apoptosis by targeting TNFRSF1B in the spleen tissues of broilers suffering from selenium deficiency. This study explored the role of selenium deficiency in apoptosis to determine the signal transduction pathways involved, and the findings will help to elucidate the biological mechanisms in which selenium is involved.

## RESULTS

### Results of microRNA profile in spleen tissues

**Figure 1:**
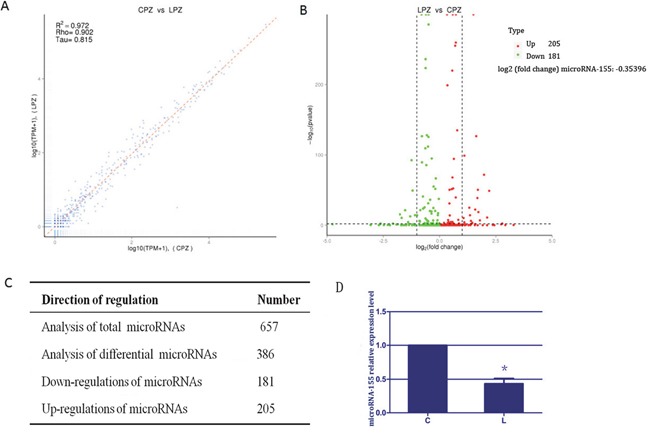
The results of microRNA expression profile and microRNA-155 in spleen tissues **(A)** represents the correlation analysis of global microRNAs expression. The scatter plot of genome-wide microRNA expression between C and L groups (Pearson r^2^ = 0.98). **(B)** indicates volcano map for different microRNA. X coordinate indicate the fold change, Y coordinate indicate the significance, red dot are up regulated microRNA, green dot are down regulated microRNA, blue dot are not different microRNA. **(C)** represents the results of all microRNAs differential analysis. Each value comes from three individuals. **(D)** exhibits the results of microRNA-155 in spleen tissue of selenium-deficient broiler. Bars with “*” indicated that there are significant differences (*P* < 0.05) between C group and L group. Each value represented the mean ± SD of five individuals.

To identify the potential microRNA signatures, we generated the expression profile of microRNA as shown in Figure 1A–1C. The comparison showed that the expression profiles of spleen tissues from the C group and L group were highly correlated (Pearson correlation, r^2^=0.972). Further examination revealed that 386 microRNAs were differentially expressed among the 657 microRNAs between the C group and L group, the levels of 205 microRNAs increased and 181 microRNAs decreased in spleen tissues of broilers due to selenium deficiency. This suggested that the microRNA profile was altered due to selenium deficiency. Additionally, the log_2_ (fold change) value for microRNA-155 was -0.35396, indicating that microRNA-155 was significantly down-regulated in the spleen tissues of selenium-deficient broilers. We assessed the expression of microRNA-155 by RT-PCR. As shown in Figure 1D, the expression of microRNA-155 in the L group was significantly reduced to ~43% (*P* < 0.05) compared with the C group. These results suggested that selenium deficiency altered the microRNA expression profile and reduced the expression of microRNA-155.

### Morphological observations of broiler spleens

**Figure 2:**
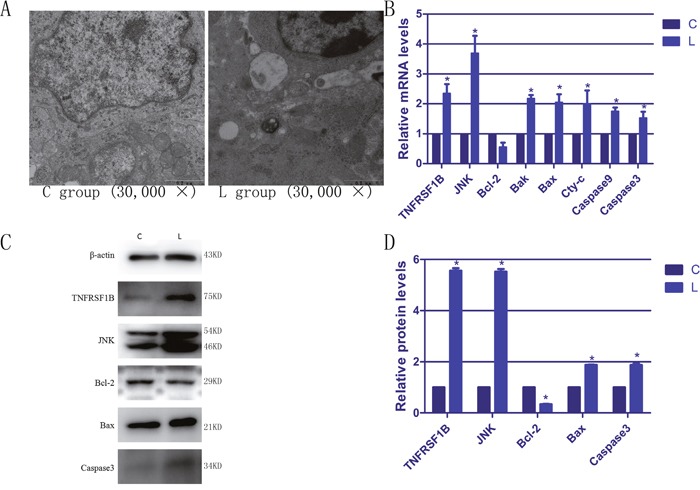
The ultrastructural changes and the expressions of apoptotic genes in spleen of selenium deficiency broiler **(A)** represents the ultrastructural change of splenic cells. **(B)** represents the mRNA expression of apoptosis genes in spleen tissues of broiler. **(C)** represents immunoblotting of β-actin, TNFRSF1B, JNK, Bcl-2, Bax and caspase3 in spleen tissues. **(D)** represents respectively the protein expression of apoptosis genes. Bars with “*” indicated that there are significant differences (*P* < 0.05) between C group and L group. Each value represents the mean ± SD of five individuals.

We used transmission electron microscopy to study apoptosis in the broiler splenic cells. As shown in Figure 2A, the spleens of broilers in the C group displayed that the normal morphologies. However, many typical apoptotic features appeared in the splenic cells of selenium-deficient broilers, including smaller cell volume, increased cytoplasmic color, vacuole formation in the cytoplasm, marked accumulation of chromatin or the formation of crescent-shaped aggregates at the nuclear membrane. All these observed features confirmed that selenium deficiency could induce apoptosis in the broiler spleen and that we had successfully reproduced the apoptotic model.

### Expression of apoptotic genes in the broiler spleens

To better understand the effects of selenium deficiency on apoptosis, we evaluated the expression of apoptotic genes by RT-PCR and WB in broiler spleens. As shown in Figure 2B–2D, we examined the expression of TNFRSF1B, JNK, Bcl-2, Bax, Bak, Cyt-c, caspase9 and caspase3 in this study. Except for Bcl-2, the expression of the rest of the genes was increased in the lymphocytes compared with the corresponding C group, and most of the differences were statistically significant (*P* < 0.05). The mRNA and protein levels of Bcl-2 were significantly reduced in comparison to the C group. These data verified that selenium deficiency could induce apoptosis in broiler spleens.

### Transfection efficiency of microRNA-155 in the splenic lymphocytes

**Figure 3:**
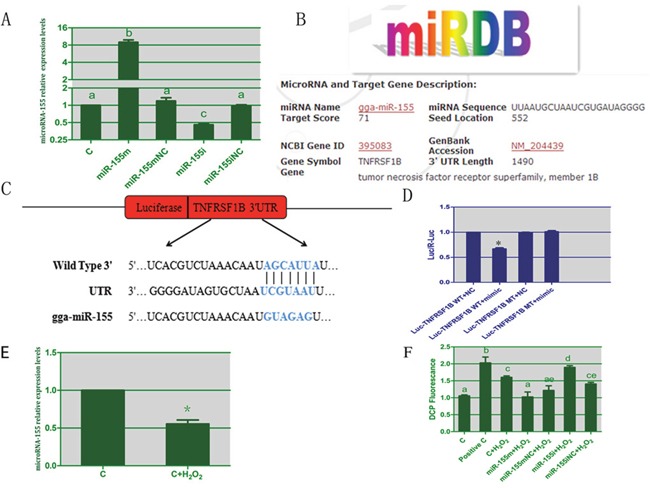
The results of TNFRSF1B as a target of microRNA-155 and the results of ROS induced by H2O2 in lymphocytes **(A)** represents the transfection efficiency of microRNA-155 in spleen lymphocytes. **(B)** represents TNFRSF1B as a predicted target of miR-155 by miRDB software. **(C)** represent the sequences of wild-type or mutant 3'UTR of TNFRSF1B and microRNA-155. **(D)** represents the results of dual luciferase reporter gene assay. **(E)** represents the levels of microRNA-155 in lymphocytes treated by H_2_O_2_. **(F)** denotes the effect of microRNA-155 on levels of ROS in lymphocytes. The different small letters indicate that there are significant differences (*P* < 0.05) between any two groups. Each value represents the mean ± SD of three individuals.

To test the efficacy of the synthetic microRNA oligonucleotides, we transfected them into cultured lymphocytes and evaluated expression of microRNA-155 by RT-PCR. As expected, compared with the C group, the level of microRNA-155 was significantly increased by 7.97 times in the microRNA-155 m group, whereas it was decreased to 47% in the microRNA-155i group as shown in Figure 3A. Thus, the microRNA oligonucleotides used in this study were effective and thus could be used for subsequent experiments.

### TNFRSF1B is a target of microRNA-155 in lymphocytes

To study the mechanism by which microRNA-155 causes apoptosis in lymphocytes, the miRDB software was used. We found that TNFRSF1B was a potential target of miR-155 as shown in Figure 3B. The TNFRSF1B 3'UTR has a binding site for microRNA-155, suggesting that TNFRSF1B may be a direct target of microRNA-155. Therefore, we tested whether the over-expression of microRNA-155 could lead to the down-regulation of TNFRSF1B expression in lymphocytes.

To understand if the relationship between microRNA-155 and TNFRSF1B was that of negative regulation, we constructed plasmids containing wild-type or mutant-type 3'UTR of TNFRSF1B fused to the luciferase gene (Figure 3C). The wild-type or mutant plasmid was co-transfected into cardiomyocytes with microRNA-155 m or microRNA-155 mNC. As shown in Figure 3D, microRNA-155 significantly decreased the luciferase activity of the wild-type 3'UTR of TNFRSF1B but not the one with the mutant 3'UTR of TNFRSF1B, suggesting that microRNA-155 could directly bind to the 3'UTR of TNFRSF1B.

### Effect of H_2_O_2_ on microRNA-155 expression and the level of ROS in lymphocytes

As shown in Figure 3E, we evaluated the effects of H_2_O_2_ on the expression of microRNA-155 in lymphocytes by RT-PCR. The microRNA-155 level was inhibited by 45% in the lymphocytes treated with H_2_O_2_ compared to the control lymphocytes. This showed that H_2_O_2_ could reduce the expression of microRNA-155 in lymphocytes.

To explore the protective effect of microRNA-155 on H_2_O_2_-induced oxidative stress in lymphocytes, we tested the levels of ROS as shown in Figure 3F. From the results of this assay, it was evident that H_2_O_2_ significantly increased the level of ROS (*P* < 0.05). In the microRNA-155 m group, the level of ROS was significantly altered in comparison with the H_2_O_2_ group. In the microRNA-155i group, the H_2_O_2_-induced ROS was increased. These data suggested that microRNA-155 had a protective effect on the lymphocytes against the H_2_O_2_-induced oxidative stress.

### Effect of microRNA-155 on apoptotic morphology

To estimate the effect of microRNA-155 on H_2_O_2_-induced apoptosis in lymphocytes, we used the TUNEL staining with DAPI and AO/EB.

**Figure 4:**
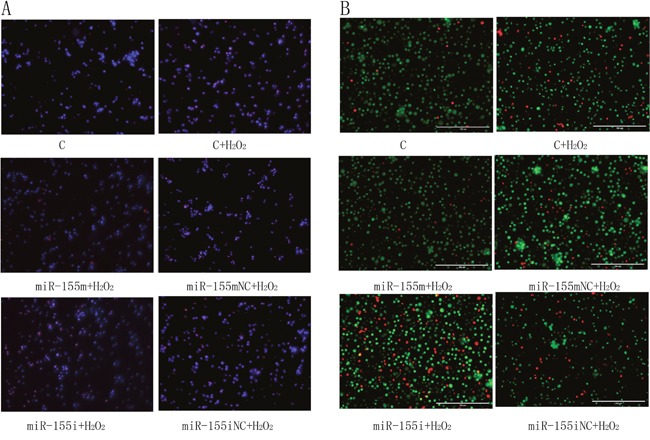
The effects of microRNA-155 on apoptotic morphology **(A)** Detection results of TUNEL staining with DAPI (magnification ×200) of the lymphocytes. Dyed blue represent normal cells. The apoptotic lymphocytes are stained by red. **(B)** Detection results of staining with AO/EB (magnification ×400) of the lymphocytes. The normal lymphocytes are stained by green. The early apoptotic lymphocytes are stained by bright green, and later apoptotic lymphocytes are stained by orange. The necrotic lymphocytes are stained by red.

As shown in Figure 4A, the apoptotic lymphocytes are represented in red. We found very few apoptotic cells in the C group. However, the H_2_O_2_-treated group displayed a higher degree of apoptosis than the C group, and microRNA-155 was able to reduce the occurrence of apoptosis induced by H_2_O_2_. However, microRNA-155i increased the severity of apoptosis, and there was no significant difference between the microRNA-155 mNC and microRNA-155iNC groups.

As shown in Figure 4B, the cells stained in light green, orange and red, represented cells undergoing early apoptosis, late apoptosis and necrosis, respectively. We observed only a few apoptotic lymphocytes in the C group. After treatment by H_2_O_2_, more cells were stained bright green, orange and red in comparison with the C group. In the microRNA-155 m group, there were more green lymphocytes than in the H_2_O_2_ group. The number of apoptotic and necrotic lymphocytes was the highest in the microRNA-155i group. These results revealed that the decreased microRNA-155 promoted apoptosis induced by H_2_O_2_ in lymphocytes.

### Effects of microRNA-155 on apoptosis in lymphocytes

**Figure 5:**
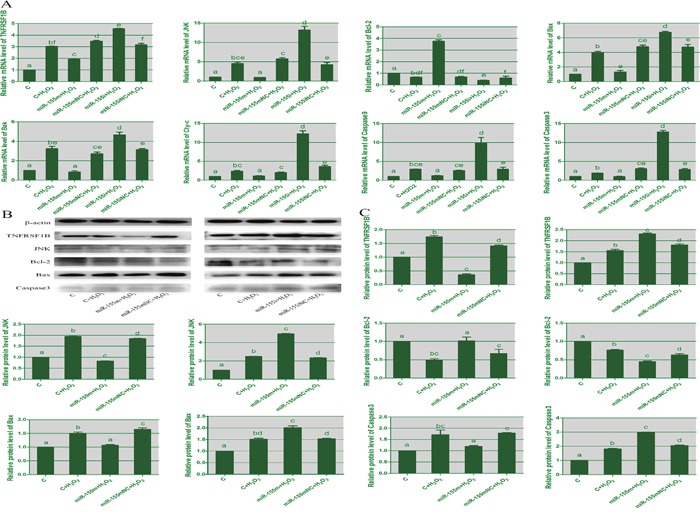
The effects of microRNA-155 on apoptosis in lymphocytes **(A)** represents the mRNA expression of apoptosis genes in lymphocytes. **(B)** represents immunoblotting of β-actin, TNFRSF1B, JNK, Bcl-2, Bax and caspase3 in lymphocytes from different groups. **(C)** represents the protein expression of apoptosis genes. The different small letters indicate that there are significant differences (*P* < 0.05) between any two groups. Each value represents the mean ± SD of three individuals.

To confirm the relation between apoptosis and microRNA-155, we evaluated the mRNA and protein levels of apoptosis-related genes in lymphocytes by RT-PCR and WB. As shown in Figure 5, when compared with the H_2_O_2_ group, in the microRNA-155i group treated with H_2_O_2_ the expression of TNFRSF1B, JNK, Bak, Bax, Cyt-c, caspase3 and caspase9 was significantly increased. This was consistent with that in the spleen tissues of selenium-deficient broilers. The levels of TNFRSF1B, JNK, Bak, Bax, Cyt-c, caspase9 and caspase3 in the microRNA-155m group, were decreased by H_2_O_2_ compared with the H_2_O_2_ group. The mRNA and protein levels of Bcl-2 were significantly reduced in the microRNA-155i group after treatment with H_2_O_2_. These data demonstrated that the reduced expression of microRNA-155 could promote H_2_O_2_-induced apoptosis in the lymphocytes.

## DISCUSSION

Selenium deficiency can lead to a variety of pathological reactions in immune organs (including spleen, thymus and bursa of Fabricius) in chickens, such as dysfunction in immune regulation, oxidative stress, inflammation caused by variants of selenoprotein genes, etc.[23–25]. However, how selenium deficiency stimulates the expression of microRNAs in broiler spleen has not been studied yet. The results from the present study indicated that selenium deficiency could alter the microRNA profile in broiler spleens. From the 657 entities included on the microRNA chip, the expressions of 386 microRNAs were found to be significantly altered following selenium deficiency. Macieldominguez et al. have reported that selenium supply could significantly affect 12 microRNAs out of 737 microRNAs in Caco-2 cells [21]. Among 621 microRNAs, only 3 microRNAs were shown to be altered by an selenium supplementation treatment in liver tissues of maturing beef [22]. The difference in the number of altered microRNAs between our results and others is probably because of the different cell types and/or different concentrations of selenium used. Among the 181 microRNAs inhibited in the present study, the expression of microRNA-155 was down-regulation due to selenium deficiency. Further analysis by RT-PCR revealed that the level of microRNA-155 was indeed reduced in the spleen tissues of broilers after selenium deficiency.

microRNA-155, an important multifunctional microRNA, has many target genes. The direct targets of microRNA-155 have been experimentally validated, including SOCS1 [26, 27], FADD, RIPK1 [28], TP53INP1 [29], MAP3K7IP2 [30], etc. In this study, we provided several lines of evidence indicating that TNFRSF1B was the target of microRNA-155 in the lymphocytes. On one hand, microRNA-155 was predicted to bind to the seed sequence of TNFRSF1B mRNA in the miRDB software. Additionally, microRNA-155 over-expression inhibited the luciferase activity of TNFRSF1B 3'UTR in wild-type, but not the mutant-type. Furthermore, the over-expression and/or knockdown of microRNA-155 respectively down-regulated and/or up-regulated the TNFRSF1B expression in the lymphocytes. Therefore, TNFRSF1B was one target of microRNA-155 in lymphocytes.

Being a TNF receptor, TNFRSF1B is expressed in a limited subset of cells such as CD4 and CD8 T lymphocytes, thymocytes, and cardiac myocytes. There are a number of studies showing that TNFRSF1B has the ability to inhibit apoptosis and activate survival pathways [31–33]. However, only a small number of reports showed that TNFRSF1B could accelerate the process of apoptosis, particularly in T cells. For example, Pimentel and Seed demonstrated that the ability of TNFRSF1B to initiate apoptosis was dependent on high levels of RIP expression in T cells [34]; Peter Vandenabeele and colleagues reported that in the rat/mouse T cell hybridoma PC60, TNFRSF1B could generate pro-apoptotic signal independently of TNFR1 [35]; Additionally, Ramesh and Reeves reported that apoptosis were reduced in TNFRSF1B-deficient mice [36]. Therefore, the expression of TNFRSF1B was used as an index for the evaluation of apoptosis in this study. This study is the first to report that the expression of TNFRSF1B mRNA and protein was induced by selenium deficiency in the broiler spleen. According to KEGG, we obtained that TNFRSF1B (TNFR2) could participate into JNK pathway to induce apoptosis, and there were studies also reported that the over-expression of TNFRSF1B indirectly mediated a signal for apoptosis by activating JNK [37, 38]. In this work, the levels of JNK mRNA and protein were increased with the increase in TNFRSF1B due to selenium deficiency. JNK, a major regulator of apoptosis, could alter the levels of the Bcl-2 family of proteins. The Bcl-2 family has both anti-apoptotic member (Bcl-2) and pro-apoptotic members (Bax and Bak) involved in the mitochondrial apoptotic signal pathway [39]. JNK can lead to Bcl-2 phosphorylation at multiple sites to promote the dissociation of the Bcl-2/Bax complex [40]. The dissociated Bax can directly induce Cyt-c release from the mitochondria [41]. Cyt-c can then activate caspase9 and the downstream signal caspase3 resulting in the mitochondrial apoptotic cascade [42–44]. Our results suggested that the up-regulation of TNFRSF1B due to selenium deficiency might indirectly stimulate JNK signaling to participate in the mitochondrial apoptotic process, but the detailed mechanisms need further study.

**Figure 6:**
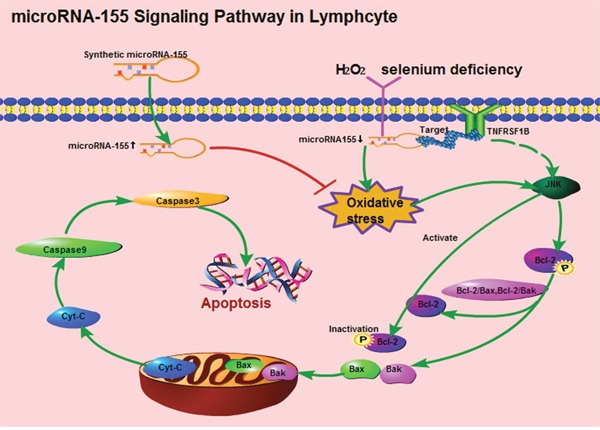
**The apoptotic signaling pathway of microRNA-155 in splenic lymphocytes**.

Selenium is thought to protect macromolecules and membrane lipids from oxidative damage by combating ROS [45], and selenium deficiency and H_2_O_2_ can increase oxidative damage and cause apoptosis [46–48]. In this study, both selenium deficiency and H_2_O_2_ reduced the expression of microRNA-155. To further ascertain whether microRNA-155 decreased oxidative stress-induced apoptosis in the splenic cells by regulating TNFRSF1B, the lymphocytes were transfected with microRNA-155 m and microRNA-155i followed by exposure to H_2_O_2_. From the results of ROS and analysis of the expression of apoptosis-related genes, we found that microRNA-155 could promote oxidative stress-induced apoptosis in the lymphocytes by activating JNK [49]. microRNA-mediated inhibition of apoptosis by binding to their targets has been reported in immune cell lines. The over-expression of microRNA-23a/b repressed apoptosis in thymic lymphoma cells by targeting Fas [50]. Transfection of microRNA-22 mimics directly targeted the galectin-9 3'UTR-suppressed apoptosis in peripheral blood mononuclear cells [51]. Additionally, Koch and Meyer have reported that microRNA-155 prevent apoptosis caused by DNA damage in macrophages [52]. We also demonstrated that the up-regulation of microRNA-155 contributed to down-regulating the expression of TNFRSF1B, which could act a protective mechanism against the oxidative stress-mediated mitochondrial apoptotic signaling pathway mediated by H_2_O_2_ in the lymphocytes. Our previous study demonstrated that selenium could induced oxidative stress in broiler spleens [24], By taking the present results into consideration, we concluded that the microRNA-155 signaling pathway in lymphocytes can be described as shown in Figure 6.

## CONCLUSIONS

In summary, the present work demonstrated that selenium deficiency could alter the microRNA expression profile and reduce the expression of microRNA-155 in broiler spleens. TNFRSF1B was found to be a target of microRNA-155 in the broiler splenic cells. The reduced microRNA-155 levels in the spleen tissues of selenium-deficient broilers might promote the oxidative stress-mediated apoptotic signaling pathway by stimulating TNFRSF1B. Our findings provide a potential novel therapeutic target for selenium deficiency-induced disease.

## MATERIALS AND METHODS

### Reagents

The following four synthetic, chemically modified short RNA oligonucleotides were purchased from Shanghai Gene Pharma Co. Ltd: microRNA-155 mimics (miR-155 m, Sense: 5’-UUAAUGCUAAUCGUGAUAGGGG-3’, Anti-sense: 5’-CCUAUCACGAUUAGCAUUAAUU-3’), microRNA-155 mimics negative control (miR-155 mNC, Sense: 5’-UUCUCCGAACGAGUCACGUTT-3’ Anti-sense: 5’-ACGUGACACGUUCGGAGAATT-3’), microRNA-155 inhibitors (miR-155i, 5’-CCCCUA UCACGAUUAGCAUUAA-3’) and microRNA-155 inhibitors negative control (miR-155iNC, 5’-CAG UACUUUUGUGUAGUACAA-3’). The Dual-Luciferase^®^ Reporter Assay System and PhRL-TK were obtained from Promega. The pMICRORNA-REPORT Luciferase vector was supplied by ThermoFisher Co. Ltd. In addition, all the nucleotides used in this work were synthetized by biotech company. Other chemicals were provided by Harbin Baijiesi Technology Co., Ltd.

### Animal treatment

All of the procedures used in the present study were approved by the Institutional Animal Care and Use Committee of Northeast Agricultural University. One hundred and fifty broilers (1-day-old; Heilongjiang Yisheng poultry Co. Ltd., Harbin, China) were randomly divided into two groups (75 chickens per group). The broilers were maintained either on a diet supplemented with selenium through the addition of 0.2 mg/kg selenium (C group)in the form of sodium selenite or on a selenium-deficient granulated diet (L group, from the selenium-deficient region of Heilongjiang Province in China, containing 0.03 mg/kg selenium). The amount of selenium used in this study was determined based on the method by Yao et al. [53]. Feed and tap water were supplied ad libitum. When the symptoms of selenium deficiency appeared in the L group at ~20 days, the broilers were euthanized by cervical dislocation, and the spleen tissues were quickly collected. The tissues were excised immediately on ice, washed in a physiological saline solution and then stored at -80°C.

### microRNA detection and analysis in spleen tissues of broiler

We extracted the total RNA from the spleen tissues of the C and L groups according to the manufacturer's instruction (Novogene Bioinformatics Technology Co., Ltd.). After quantification and qualification of the total RNA, the RNA was used as input material for the synthesis of small RNA libraries. The sequencing libraries were generated using NEBNext^®^ Multiplex Small RNA Library Prep Set for Illumina^®^ (NEB, USA) following the manufacturer's recommendations, and index codes were added to attribute the sequences to each sample. Raw data (raw reads) in fastq format were first processed through custom Perl and Python scripts. Bioinformatics analysis on the differential data from the of the two groups were performed using the DESeq R package. Corrected *P*-value of 0.05 was set as the threshold for significant differential expression by default.

### Ultrastructural observations

The spleen tissues were fixed with 2.5% glutaraldehyde and rinsed twice for 15 min in 0.2 M phosphate buffer (pH 7.2). The samples were post-fixed in 1% buffered osmium tetroxide for 1 h, dehydrated through a graded alcohol series and embedded in epoxy resin. Ultrathin sections were stained with uranyl acetate before examination under a transmission electron microscope [54].

### Cell culture and transfection

Spleens were collected aseptically from the broilers (40-day-old) and placed in sterile phosphate-buffered saline (PBS; 0.1 mol/L phosphate buffer with 0.85% NaCl, pH 7.2). Single cell suspensions were prepared by gently pushing the splenic pulp through sterile stainless steel mesh with a pore size of 200 μM. The cells were washed and resuspended in 5 mL sterile PBS and layered over 5 mL lymphocyte separation medium (TianJin Haoyang Biological Manufacture Co. Ltd., China). The splenocyte preparations were enriched by centrifugation (2,000×g) for 15 min at room temperature. The cells were recovered from the interface, resuspended, and washed twice in PBS. The cells were suspended in RPMI-1640 medium [containing 10% fetal bovine serum (FBS, Gibco, USA), and 1% antibiotic-antimycotic solution (Sigma, USA)]. The splenic lymphocyte density was adjusted to 1.5 × 10^6^ cells/mL, and the viability of the freshly isolated cells was always above 95%.

Transfection reactions were carried out using Lipofectamine 2000 transfection reagent (Invitrogen), following the manufacturer's instructions. The lymphocytes were transfected with various concentration of microRNAs using 2 μL Lipofectamine 2000 in Opti-MEM medium. For the monitoring the various parameters in the present investigation, the cells were treated for 24 h in the absence and presence of 20 μM H_2_O_2_ (the process of cell culture, transfection and the concentration of H_2_O_2_ were based on the study by Yu et.al,. [55, 56]).

### Dual luciferase reporter assay

The pMIR-REPORT plasmids for the microRNA-155 target TNFRSF1B 3'UTR were constructed as wild-type (WT) pMIR-TNFRSF1B containing two tandem repeats of microRNA-155 response elements from TNFRSF1B 3'UTR or as mutant (MUT) pMIR-TNFRSF1B. The sequences of the single-stranded oligo pairs used to generate the pMIR-TNFRSF1B (WT and MUT). The oligonucleotides were annealed and inserted into the pMIR-REPORT vector (Thermo Fisher). The empty vector (pMIR-REPORT) was used as the negative control. Cardiomyocytes were seeded in 24-well plates, and cells in each well were co-transfected pMIR-TNFRSF1B and miR according to the manufacturer's protocol. Twenty-four hours after transfection, luciferase activity was measured with the Dual Luciferase Assay System (Promega). The activity of Renilla luciferase was normalized to the activity of Firefly luciferase (Renilla LUC/ Firefly LUC).

### Determination of ROS generation in lymphocytes treated with H_2_O_2_

The levels of ROS were measured using a detection kit from Beyotime Institute of Biotechnology (Nantong, China) according to the manufacturer's instructions. Next, fluorescence distribution of the lymphocytes was detected by fluorospectrophotometer analysis at an excitation wavelength of 488 nm and at an emission wavelength of 525 nm.

### TUNEL assay

For TUNEL assay, the lymphocytes were collected after treatment. The TUNEL assay was performed to detect DNA fragmentation indicative of cell apoptosis with a commercial cell apoptosis detection kit (Roche, USA) according to the manufacturer's protocol. The apoptotic cells were photographed with an Inverted microscope (DMI4000, Leica, Germany) at 200× magnification.

### AO/EB-stained

Apoptosis was determined morphologically after staining the cells with AO/EB followed by fluorescence microscopy inspection. After being transfected with microRNA-155 and treated with H_2_O_2_, the lymphocytes were stained with AO/EB according to the manufacturer's instructions (Beyotime, Beijing, China).

### RNA isolation and real-time PCR (RT-PCR)

For the quantification of microRNA-155 and mRNA of target genes by RT-PCR, total microRNANA and total RNA were isolated from the tissues and cultured cells. Reverse transcription was performed with the miRcute microRNA First-Strand cDNA Synthesis Kit (Tiangen Biotech Co. Ltd., Beijing) according to the manufacturer's instructions (Roche).

The primers for the detection of microRNA-155 and target mRNA by RT-PCR are shown in Table 1. RT-PCR was performed on a LightCycler^®^ 480 II Detection System (Roche). Reactions were performed according to manufacturer's instructions (Tiangen Biotech Co. Ltd., and Roche). U6 and β-actin served as the endogenous controls for normalization. The expression of microRNA-155 and target mRNAs relative to U6 and β-actin, respectively, were calculated using the 2^-ΔΔCT^ method [57]. We calculated the results with the following formulae: Ratio=2^-ΔΔCt^, ΔΔCt=(Ct_target_−Ct_β-actin_)_Sample_-(Ct_target_- Ct_β-actin_)_Control_

**Table 1:** microRNAs and mRNAs primer sequences

**A**		
**Gene**	**Forward primer (5’-3’)**	**Reverse primer (5’-3’)**
miR-155	GGCTTAATGCTAATCGTGATAGGGGT	Provided by Tiangen Biotech Co. Ltd.
U6	CACGCAAATTCGTGAAGCGTTCCA	Provided by Tiangen Biotech Co. Ltd.
**B**			
**Genes**	**Serial number**	**Forward primer (5’-3’)Reverse primer (5’-3’)**	**Product size (bp)**
TNFRSF1B	XM_015296812	F:TGCCTACTCACAGCCAACTGR:AGATGCTGCTCCTCCTGTTC	126
JNK	XM_015288440.1	F:GCATCCATCTTCGTCGTCATR:TCATCTACAGCAACCCAGAGG	121
Bcl-2	D11382.2	F:ATCGTCGCCTTCTTCGAGTTR:ATCCCATCCTCCGTTGTCCT	150
Bak	NM_001030920.1	F:ACCCGGAGATCATGGAGAR:GATGCCTTGCTGGTAGACG	272
Bax	XM_422067.2	F:GTGATGGCATGGGACATAGCTCR:TGGCGTAGACCTTGCGGATAA	90
Cty-c	K02303.1	F: AGGCAAGCACAAGACTGGAR: CTGACTATCACCAAGAACCACC	150
caspase3	NM_204725.1	F:CTGAAGGCTCCTGGTTTAR:TGCCACTCTGCGATTTAC	104
caspase9	XM_424580.3	F:CCGAAGGAGCAAGCACGR:AGGTTGGACTGGGATGGAC	243
β-actin	L08165	F:CCGCTCTATGAAGGCTACGCR:CTCTCGGCTGTGGTGGTGAA	128

A represents microRNAs primer sequences; B represents mRNAs primer sequences.

### Protein extraction and western blot (WB) analysis

The protein samples were separated by 12% SDS-PAGE and transferred to PVDF membranes. The membranes were blocked with 5% skim milk for 12 h and incubated for 1 h at 37°C with the following diluted primary antibodies: TNFRSF1B (1/100), c-Jun N-terminal kinase (JNK) (1/100), caspase3 (1/500), Bax (1/500) and Bcl-2 (1/100). After washing four times for 5 min each with PBST, the membranes were incubated for 1 h at 37°C with peroxidase-conjugated secondary antibodies against rabbit IgG (1/2000, Santa Cruz, USA). After washing four times for 5 min each, the bound antibodies were visualized by chemiluminescence using the ECL-plus reagent (GE Healthcare, Buckinghamshire, UK). The β-actin content was analyzed as the loading control with rabbit polyclonal antibody (Sigma, USA).

### Statistical analyses

Statistical analyses of all data were performed using GraphPad Prism (version 5.0, GraphPad Software Inc., San Diego, CA, USA). The significant values (*P* < 0.05) were obtained by one-way ANOVA. All data displayed normal distribution and passed the test for equal variance. The data are expressed as the mean ± SD, and the differences were considered to be significant if *P* < 0.05.

## Abbreviations

RT-PCR: real-time PCR
WB: Western blot
TNFRSF1B: tumor necrosis factor receptor superfamily member 1B
miR-155m: microRNA-155 mimics
miR-155mNC: microRNA-155 mimics negative control
miR-155i: microRNA-155 inhibitors
miR-155iNC: microRNA-155 inhibitors negative control
JNK: c-Jun N-terminal kinase.
